# Reducing stunting in India: what investments are needed?*

**DOI:** 10.1111/mcn.12291

**Published:** 2016-05-17

**Authors:** Rasmi Avula, Neha Raykar, Purnima Menon, Ramanan Laxminarayan

**Affiliations:** ^1^ International Food Policy Research Institute (IFPRI) New Delhi India; ^2^ Public Health Foundation of India (PHFI) New Delhi India

India has among the highest rates of child malnutrition rates in the world, but these rates have been declining rapidly during the past decade. Between 2006 and 2014, stunting rates for children under five in India have declined from 48 to 38% (Global Nutrition Report, 2014). Despite this progress, child undernutrition rates in India are among the highest in the world, with nearly one‐half of all children under 3 years of age being either underweight or stunted. India is still home to over 40 million stunted children and 17 million wasted children (Global Nutrition Report, 2014). In addition, the rates of decline have been highly variable across India's states. Some states, including Arunachal Pradesh, Mizoram and Delhi, had large rates of reduction in stunting, but overall levels of undernutrition remained high because of high baseline rates. Meanwhile, in Uttar Pradesh, Jammu and Kashmir, Manipur and Jharkhand the situation has not changed significantly (Raykar *et al.,* 2015). Similar variability is observed in the prevalence of anaemia rates as well, which range from 38% in Goa to 78% in Bihar (IIPS & Macro International, International Institute for Population Sciences (IIPS) and Macro International, 2007).

Global evidence shows that child malnutrition is only weakly correlated with income. In fact, a quarter of Indian children from the top income quintile were stunted in 2006. Stunting is a marker for poor environmental, maternal and child factors, including poor sanitation, intrauterine growth restriction, micronutrient deficiencies, and sub‐optimal infant and young child feeding practices. Current global recommendations for achieving 20% reduction in stunting and 61% reduction in severe wasting include delivery of a set of nutrition‐specific interventions at 90% coverage level (Bhutta *et al.,* 2013). These interventions span the continuum of care and include food and micronutrient supplements before and during pregnancy, counselling for initiation of breastfeeding and food and micronutrient supplementation for mothers in the newborn period and breastfeeding counselling, food and micronutrient supplementation along with routine immunization for the under five children (Fig. 1). Available data indicate that less than 50% of mothers and children in India are exposed to a majority of these interventions. The shortfall is greater for iron folic acid supplementation, food supplementation and minimum diet diversity, whereas exclusive breastfeeding and immunization have improved in recent years (Fig. 2).

**Figure 1:**
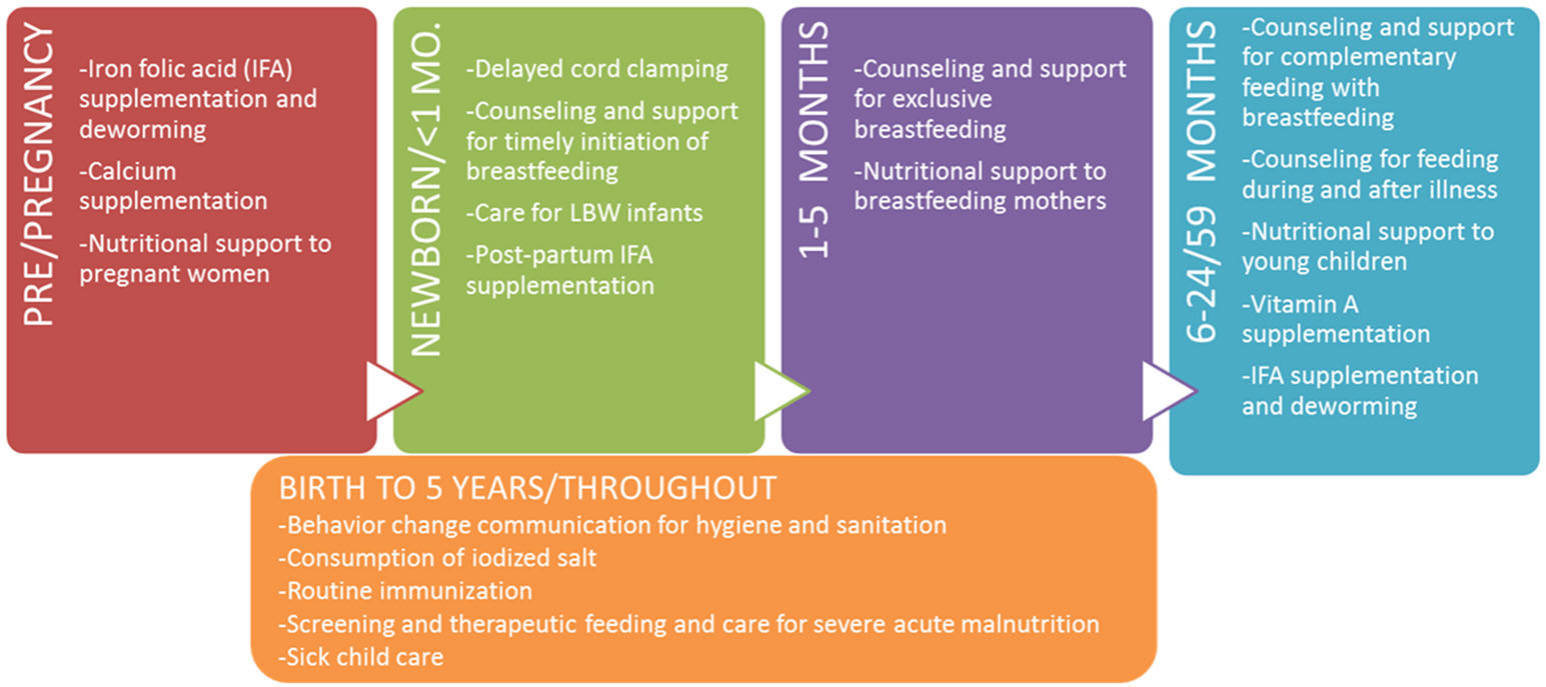
Continuum of care.

**Figure 2:**
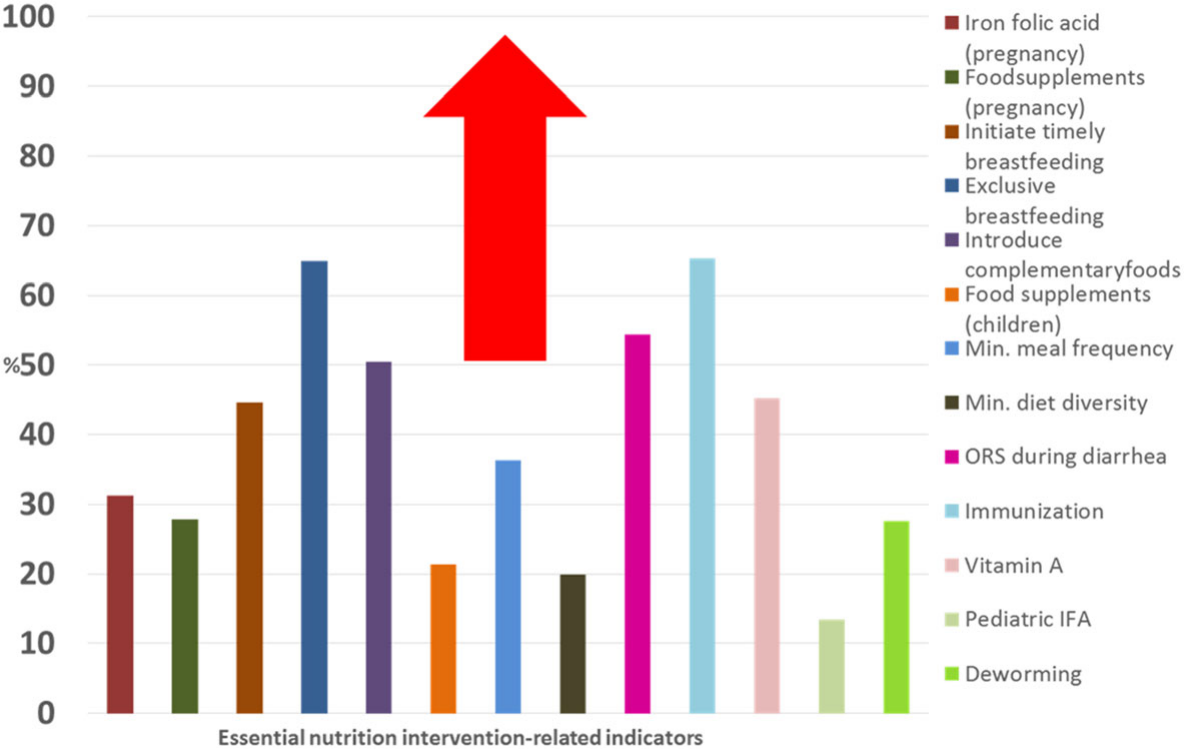
Major gaps in the delivery of essential nutrition interventions (ENIs) in India Ministry of Women and Child Development (2015).

## Policy and programmatic context, implementation guidance and financing

In India, the policy and programme environment to facilitate at‐scale delivery of nutrition‐specific interventions is broadly in place but varies by intervention. Supportive policies exist (except for community‐based management of acute malnutrition, for which guidelines are under development) (Vir *et al.,* 2013). However, operational guidelines and/or monitoring indicators are only available for a few of the interventions (e.g. monitoring indicators do not exist for counselling for infant and young child feeding practices; guidelines are under development for paediatric iron folic acid supplementation). Two national programmes, implemented by separate ministries, Ministry of Women and Child Development and Ministry of Health and Family Welfare, operate across the country and are designed to *together* deliver all of the nutrition‐specific interventions through their frontline workers (Avula *et al.,* 2013). There are, however, gaps in the implementation of these interventions, arising from challenges such as operationalizing interministerial convergence, limited understanding of the frontline worker roles and responsibilities, inadequate training, limited mechanisms for supportive supervision, and the burden of multiple responsibilities for frontline workers and other health staff. In addition to these challenges, operational evidence for at scale implementation of all nutrition‐specific interventions, especially across the continuum of care, is limited. There are few published studies and a limited grey literature on *how* to deliver nutrition‐specific interventions in ways that achieve impact, specifically for interventions on complementary feeding counselling, prevention of paediatric anaemia, and treatment of severe and acute malnutrition (Avula *et al.,* 2013).

From a financing perspective, India requires at least $6bn annually to deliver the nutrition‐specific interventions at full coverage. A bulk of this amount is required for food supplementation (39%), while counselling, health interventions and micronutrient supplementation and deworming together require much less – 12% of the total cost (Menon *et al.,* 2015). The cost of implementing the interventions varies tremendously by the state and is driven by the target population base of the state. Thus, populous states like Uttar Pradesh require more funds (close to $1bn) for delivering the interventions compared with states like Kerala or Chhattisgarh. A challenge for India, looking ahead, is managing fiscal devolution appropriately for health and nutrition to ensure that at the state‐level, adequate financing will continue to be available, along with capacity to manage the delivery of nutrition interventions across the continuum of care.

## Underlying determinants of nutrition and nutrition‐sensitive interventions

Several factors influence stunting other than those addressed through nutrition‐specific interventions. Antenatal care, decreased open defecation, low fertility, agriculture, safe water and sanitation, women's education and empowerment, and the quantity and quality of food available have been the key drivers of stunting reductions, while income growth and governance played a facilitating role (Headey *et al.,* 2014; Smith & Haddad, 2014). Nearly 55% of the population in India defecates in the open, which puts them at greater risk of enteric diseases (Ministry of Women and Child Development, 2014), and only 12% of women have secondary school education (Ministry of Home Affairs. Census, 2011). To achieve accelerated reductions in stunting, increased investments in programmes that address these underlying determinants are imperative (Ruel *et al.,* 2013). Poverty, food insecurity, early marriages, high fertility rates and birth spacing have to be addressed to achieve greater reductions in stunting.

India has several social safety net programmes that aim to enhance food security and bridge gaps in seasonal availability of work. The national food security act aims to extend coverage of consumer food subsidy, provide subsidized grains and other essential commodities through the Public Distribution System. The supplementary nutrition programme of the Integrated Child Development Services aims to bridge the calorie and nutrient gap among pregnant and lactating women and children under 6 years, while the Mid‐Day Meal scheme attempts to do the same for school children. The National Rural Employment Guarantee Scheme aims to provide work to ensure resources at home in rural areas during non‐agriculture seasons. Additionally, there is a national campaign, the *Swachh Bharat Abhiyaan*, to improve sanitation facilities and their usage through providing support for building toilets. Across the states, however, these programmes are fraught with different implementation challenges such as poor targeting, leakages, inadequate infrastructure, corruption and delayed payments, and this is reflected in the state of these underlying determinants (Raykar *et al.,* 2015). It is timely, therefore, to revisit programme designs and to identify and test new approaches to plug implementation gaps if these large‐scale social safety net programmes are expected to influence the underlying determinants of stunting.

## The way forward

India has several existing policies and programmes, both nutrition‐specific and nutrition‐sensitive to address several known drivers of stunting, other dimensions of nutrition and child development. However, the utility and impact of these programmes will vary across India due to gaps in the implementation of the policies and programmes. Gaps in implementation are often a result of complicated design of the programmes, lack of operational guidelines, limited financial and human resources for adequate and high quality implementation, poor monitoring and lack of political commitment at the state level. There is a need for focused efforts on closing implementation gaps and building evidence, revisiting programme designs, and establishing feedback mechanisms to inform policy and programmatic decisions.

In conclusion, an all‐out effort is now needed to improve the functioning of nutrition‐specific interventions as well as a focus on addressing underlying social factors by reducing income inequality, improving the health and social status of women, scaling‐up water and sanitation services, and addressing food insecurity. The most critical aspect is to ensure that all of these multiple investments converge at the same time, at the same place, for the same mother–child dyad. India's birth cohort of 27 million babies each year deserve better life conditions than those that await them at present (Raykar *et al.,* 2015).

## Source of funding

POSHAN (*Partnerships and Opportunities to Strengthen and Harmonize Actions for Nutrition in India)* was funded by the Bill & Melinda Gates Foundation, and *Transform Nutrition* was funded by UKAid. Neither funding agency played any role in the development of this perspective piece*.*

## Contributions

RA and PM drafted the commentary. NR and RL revised the draft. All the authors read the final commentary and approved it.

## Conflicts of interest

The authors declare that they have no conflicts of interest.
